# Reduced cortical complexity in ventromedial prefrontal cortex is associated with a greater preference for risky and immediate rewards

**DOI:** 10.1162/imag_a_00143

**Published:** 2024-04-18

**Authors:** Fredrik Bergström, Caryn Lerman, Joseph W. Kable

**Affiliations:** Faculty of Psychology and Educational Sciences, University of Coimbra, Portugal; Department of Psychology, University of Gothenburg, Sweden; Department of Psychiatry, Perelman School of Medicine, University of Pennsylvania, Philadelphia, PA, United States; Department of Psychology, University of Pennsylvania, Philadelphia, PA, United States

**Keywords:** risky choice, intertemporal choice, cortical complexity, ventromedial prefrontal cortex, magnetic resonance imaging (MRI), brain structure

## Abstract

In our everyday lives, we are often faced with situations in which we make choices that involve risky or delayed rewards. However, the extent to which we are willing to accept larger risky (over smaller certain) or larger delayed (over smaller immediate) rewards varies across individuals. Here, we investigated the relationship between cortical complexity in the medial prefrontal cortex and individual differences in risky and intertemporal preferences. We found that reduced cortical complexity in left ventromedial prefrontal cortex (vmPFC) was associated with a greater preference for risky and immediate rewards. In addition to these common structural associations in left vmPFC, we also found associations between lower cortical complexity and a greater preference for immediate rewards that extended into the left dorsomedial prefrontal cortex and right vmPFC. Furthermore, these structural associations occurred in a context where a preference for risky rewards was correlated with a preference for delayed rewards across individuals. These results suggest that risk and intertemporal preferences are distinct but related, and likely influenced by multiple neurocognitive processes, with cortical complexity in vmPFC reflecting one shared aspect possibly related to impulsiveness in terms of risky and impatient economic choice. Future work should elucidate the complex relationships between brain structure and behavioral preferences.

## 1 Introduction

In our daily lives, we frequently make decisions that involve risky or delayed rewards. As individuals, we differ in our tendency to accept larger risky (over smaller certain) or larger delayed (over smaller immediate) rewards. An ongoing research question is how differences in risky and intertemporal preferences are related to each other and to differences in brain structure (for a review, seeKable & Levy, 2015). Cortical surface complexityYotter, Nenadic, et al. (2011)is a structural property relatively more rooted in genetics and early neurodevelopment (Armstrong et al., 1995;Kochunov et al., 2010;Zilles et al., 1988), and may therefore be well suited to explain trait differences in choice behavior. The ventromedial prefrontal cortex (vmPFC) is central to estimating the subjective value of risky or delayed rewards (Bartra et al., 2013;Kable & Glimcher, 2009), and lesions to vmPFC disrupt the valuation process (Camille et al., 2011;Fellows & Farah, 2007;Yu et al., 2022) and lead to a greater preference for risky and immediate rewards (Mok et al., 2021;Peters & D’Esposito, 2020;Sellitto et al., 2010). Here, we looked for a relationship between cortical complexity in the vmPFC and preferences for risky and immediate rewards, in a sample of healthy human participants.

Several hypotheses have proposed potential links between risk and intertemporal preferences. For example, the “delay-as-risk hypothesis” posits that longer delays naturally imply more uncertainty and so individuals who show a greater preference for risky rewards should also prefer delayed rewards (Benzion et al., 1989;Keren & Roelofsma, 1995). Similarly, the “cognitive ability hypothesis” posits that higher cognitive ability leads to a greater preference for (calculated) risky and delayed rewards (Burks et al., 2009). Contrarily, the “impulsivity hypothesis” posits that a tendency towards impulsive choices should lead towards a greater preference for both risky and immediate rewards (Holt et al., 2003;Myerson et al., 2003). However, empirically, risk and intertemporal preferences are only weakly to moderately related to each other, usually such that a greater preference for risky and delayed rewards goes together (for a meta-analysis, seeBurks et al., 2009;Holt et al., 2003;Johnson et al., 2020;Myerson et al., 2003). This relationship is in the opposite direction from what impulsivity predicts and too weak for uncertainty discounting or cognitive ability to be the sole driver of both preferences. Thus, risk and intertemporal preferences are likely determined by some distinct and (possibly) some shared cognitive processes. Accordingly, one would expect that there are both distinct and shared brain mechanisms that together produce risk and intertemporal preferences, and potentially multiple shared mechanisms that impart differently signed correlations between the two.

Many brain areas are involved in risky and intertemporal choices (for reviews, seeKable & Levy, 2015;Knutson & Huettel, 2015;Mohr et al., 2010;Peters & Büchel, 2011), but the vmPFC plays a key role in both. Neural activity in vmPFC and anterior ventral striatum (aVS) reflects domain-general subjective valuation processes (Bartra et al., 2013;Kable & Glimcher, 2009), and activity in these regions correlates with the value of risky or delayed rewards after incorporating individual tolerance levels for risk (Levy et al., 2010) or delay (Kable & Glimcher, 2007). Correspondingly, lesions to vmPFC disrupt the stability of valuations and lead to inconsistent choice (Camille et al., 2011;Fellows & Farah, 2007;Yu et al., 2022). Studies on the effects of lesions to vmPFC on risky and intertemporal choice have found mixed results for risky (Clark et al., 2008;Leland & Grafman, 2005;Levens et al., 2014;Manes et al., 2002;Spaniol et al., 2019;Studer et al., 2015;Weller et al., 2007) and intertemporal (Fellows & Farah, 2005;Mok et al., 2021;Peters & D’Esposito, 2016;Sellitto et al., 2010) choice separately, but overall tend towards lesions causing greater preference for risky and immediate rewards (for a review, seeYu et al., 2020). Importantly, the two studies that examined the effects of vmPFC lesions on risky and intertemporal choice in the same individuals both found a greater preference for risky and immediate rewards (Mok et al., 2021;Peters & D’Esposito, 2020).

To date, studies have tried to link brain structure to risky or intertemporal preferences separately, and focused on grey matter volume (GMV) and cortical thickness. The findings are quite varied but some studies have found relationships with structural properties of the medial prefrontal cortex (mPFC). A greater preference for immediate rewards has been positively associated with more GMV in mPFC, posterior cingulate cortex (pCC), middle temporal cortex, and entorhinal cortex; reduced GMV in dorsolateral prefrontal cortex (dlPFC), inferior frontal cortex (IFC), and superior frontal gyrus; both more and reduced GMV in the putamen (Bjork et al., 2009;Cho et al., 2013;Owens et al., 2017;Schwartz et al., 2010); and reduced cortical thickness in mPFC (Bernhardt et al., 2014;Drobetz et al., 2014;Pehlivanova et al., 2018), entorhinal cortex (Lempert et al., 2020), temporal pole, and temporoparietal junction (Pehlivanova et al., 2018). A greater preference for risky rewards has been associated with more GMV in amygdalae (Jung et al., 2018), right posterior parietal cortex (PPC) (Gilaie-Dotan et al., 2014;Grubb et al., 2016), and cerebellum (Quan et al., 2022).

Cortical complexity is a measure of how space-filling, self-similar, and convoluted a brain’s surface isYotter, Nenadic, et al. (2011), and high cortical complexity has been associated with thinner cortex, deeper sulci, higher folding frequency, and more convoluted gyral shape (Im et al., 2006;Jiang et al., 2008;King et al., 2010). The structural properties underlying cortical complexity are deeply rooted in genetics and early neurodevelopment, and may therefore be temporally more stable in adults than GMV (Armstrong et al., 1995;Kochunov et al., 2010;Zilles et al., 1988), but are sensitive to age-related atrophy (Madan & Kensinger, 2016). Although individual preferences change due to age, environment, and temporary fluctuations, both risk and intertemporal preferences are relatively stable over time as demonstrated by moderately high rank-order stability across individuals, suggesting that risk and intertemporal preferences are relatively stable traits (Chuang & Schechter, 2015;Escobar et al., 2023;Hertwig et al., 2019;Mata et al., 2018;Meier & Sprenger, 2015;Seneca et al., 2012;Zeynep Enkavi et al., 2019). Although cortical complexity has mainly been used to study clinical markers (e.g.,Hedderich et al., 2020;King et al., 2010;Nenadic et al., 2014,^2017^), sex differences (Luders et al., 2004,^2006^), and differences in intelligence (Hedderich et al., 2020;Im et al., 2006), if risk and intertemporal preferences are relatively stable traits, then it is plausible that there could be associations between cortical complexity and these individual preferences.

Here, we investigated the relationship between cortical complexity and individual differences in risk and intertemporal preferences in a healthy sample. We focused on mPFC as a region of interest given its role in risky and intertemporal choice, and previous work showing that lesions to vmPFC (extending to the greater mPFC area) led to a greater preference for risky and immediate rewards. We found that lower cortical complexity in left vmPFC was associated with a greater preference for risky and immediate rewards.

## 2 Materials and Methods

### 2.1 Participants

In this study, we used previously published data acquired for the Retraining Neurocognitive Mechanisms of Cancer Risk Behavior (RNMCRB) study (Kable et al., 2017). RNMCRB participants were randomized to receive 10 weeks of adaptive cognitive training (Lumosity games) or non-adaptive, untargeted cognitive stimulation (simple computerized video games) and underwent pre- and post-intervention brain scans. The study acquired high-resolution T1-weighted anatomical MRI, resting-state fMRI, diffusion tensor imaging, and task-based fMRI during a risky and an intertemporal choice task. Participants were excluded if they had a history of brain injury, a history of psychiatric or substance disorders, current use of psychotropic medication, current use of chewing tobacco, snuff, or smoking cessation products, left-handedness, intellectual disability (<90 score on Shipley’s Intelligence Quotient (IQ) Test), or had a risk tolerance α < 0.34 or α > 1.32 or discount rate k < 0.0017 or k > 0.077 (to avoid ceiling and floor effects). All study procedures were approved by the Institutional Review Board of the University of Pennsylvania, and all participants provided written informed consent. The main trial outcomes have been described in a previous report, but found no effect of cognitive training relative to active control on brain activity, decision-making, or cognitive performance (Kable et al., 2017).

For this study we used 166 anatomical high-resolution T1-weighted images and choice data from a risky and intertemporal task collected in the MRI scanner during the pre-intervention baseline session ofKable et al. (2017). We excluded nine participants for whom it was impossible to reliably estimate risky or intertemporal preference because of one-sided choices. Additionally, we quality-checked the cortical complexity image quality in CAT12 by performing overall correlations between all participants (to check sample homogeneity) and found that five participants had an overall correlation value reduced by more than two standard deviations from the median. At closer inspection, one of the participants had an artifact in the left vmPFC and was therefore excluded. We therefore used 156 participants in our analyses (90 male, 66 female; education, 3.8% grade school or some high school, 13.5% high school graduate or GED, 28.2% some college or technical school, 54.5% college graduate or beyond; age, M = 25, SD = 6 years; IQ, M = 111, SD = 7; intertemporal preference (log10(k)), M = -1.74, SD = 0.36; risk preference (log10(α)), M = -0.20, SD = 0.17).

### 2.2 Risky choice task

In the risky choice task, participants had to make a choice between receiving a smaller but certain reward (i.e., 100% probability to receive $20) or a larger but riskier reward (e.g., 47% probability to receive $84) on 120 trials. The certain reward was always fixed at $20 on all trials, while the probability and risky reward amount varied across trials. Each trial began by presenting the probability and reward amount for the risky alternative (the fixed certain option was never displayed). Participants had 4 s to accept or reject the risky alternative, after which a marker indicating their choice (a checkmark if the risky alternative was accepted, and “X” if rejected) appeared for 1 s. We estimated individual risk tolerance (α) by fitting the following logistic function to our choice data using maximum likelihood estimation (goodness-of-fit: M_R2_= 0.37, SE_R2_= 0.01) with function minimization routines (fmincon) in MATLAB (for code see,https://github.com/sangillee/UMm):

$$P1=\frac{1}{1+exp\left(-\beta \left(SV1-SV2\right)\right)},P2=1-P1$$

where P1 refers to the probability the risky option was chosen and P2 the probability that the safe option was chosen. SV1 refers to the subjective value of the risky option and SV2 to the subjective value of the safe option. β refers to a scaling factor that was fitted for each subject. The SV of the choice options was assumed to follow a power utility function:

$$SV=p\times A^{\alpha }$$

where p is the probability of winning amount A, and α is a risk tolerance parameter that was fitted for each participant. For the risky option, there is always a 1 - p chance of not winning anything. Higher α indicates higher risk tolerance or lower risk aversion.

### 2.3 Intertemporal choice task

In the intertemporal choice task, participants had to make a choice between receiving a smaller but immediate reward (i.e., receiving $20 today) or a larger but delayed reward (e.g., receiving $40 in 31 days). The immediate reward was always fixed at $20 on all trials, while the delay time and delayed reward amount varied across trials. Each trial began by presenting the delay time and reward amount of the delayed option (the fixed immediate option was never displayed). The participants had 4 s to accept or reject the delayed alternative, after which a marker indicating the choice (a checkmark if the delayed alternative was accepted, and “X” if rejected) appeared for 1 s. We estimated individual delay discount rate (k) by fitting the following logistic function to our choice data using maximum likelihood estimation (goodness-of-fit: M_R2_= 0.57, SE_R2_= 0.01) with function minimization routines (fmincon) in MATLAB:

$$P1=\frac{1}{1+\text{exp}\left(-\;\beta \left(SV1-SV2\right)\right)},P2=1-P1$$

where P1 refers to the probability the delayed option was chosen and P2 the probability that the immediate option was chosen. SV1 refers to the subjective value of the delayed option and SV2 to the subjective value of the immediate option. β refers to a scaling factor that was fitted for each subject. The SV of the choice options was assumed to follow hyperbolic discounting:

$$SV=\frac{A}{1+kD}$$

where A is the amount of the delayed option, D is the delay time until receiving the reward (for immediate choice, D = 0), and k is a discount rate parameter that was fitted for each participant. Higher values of k indicate reduced tolerance for delay or more impatience.

Both the risky and intertemporal choice tasks were incentive compatible. At the end of the experiment, participants received the option they chose on one randomly selected trial across both choice tasks. If the randomly selected trial was from the risky choice task and the participant chose the risky option, the gamble was resolved by the roll of a die. If the randomly selected trial was from the intertemporal choice task and the participant chose the delayed option, they only received the delayed amount after the number of days specified on that trial. All payments were delivered on a prepaid debit card given to the participant.

### 2.4 MRI acquisition

High-resolution T1-weighted anatomical images were acquired by a Siemens 3T Trio scanner (Siemens, Erlangen, Germany) using a magnetization-prepared rapid gradient echo (MPRAGE) sequence [repetition time (TR) = 1630 ms, echo time (TE) = 3.11 ms, voxel size = 0.94 x 0.94 x 1.0 mm, 160 axial slices, 192 x 256 matrix].

### 2.5 Preprocessing of MRI data

We used the default processing pipeline of the Computational Anatomy Toolbox version r1743 (CAT12;Gaser & Dahnke, 2016) for SPM12 (Welcome Trust Centre for Neuroimaging, London, UK), on MATLAB R2019a (Mathworks, Inc., Sherborn, MA, USA), to process the anatomical T1-weighted images and extract local cortical surface complexity (i.e., fractal dimension), gyrification, sulci depth, cortical thickness, and GMV.

For voxel-based preprocessing, T1-weighted images underwent spatial adaptive non-local means (SANLM) denoising filter (Martı & Manjo, 2010), were internally resampled, bias corrected, and affine-registered, followed by standard SPM unified tissue segmentation into grey matter, white matter, and cerebral spinal fluid (Ashburner & Friston, 2005). The resulting brain images were then skull-stripped, parcellated into left and right hemispheres, subcortical areas, and cerebellum; subject to local intensity transformation of all tissue classes to reduce effects of higher grey matter intensities before the final Adaptive Maximum A Posteriori (AMAP) tissue segmentation (Rajapakse et al., 1997), and refined by a partial volume estimation (Tohka et al., 2004). The GMV maps were spatially registered to a volumetric MNI template, resampled to 1.5 mm^3^, and spatially smoothed with an 8 mm Gaussian FWHM kernel.

For surface-based preprocessing, a projection-based thickness method was used to estimate and reconstruct cortical thickness of the central surface (Dahnke et al., 2013), after which topological correction was applied to repair defects with spherical harmonicsYotter, Dahnke, et al. (2011), and surface refinement. The final central surface mesh was used to estimate local cortical surface complexity (i.e., fractal dimension) values based on spherical harmonic reconstructionsYotter, Nenadic, et al. (2011). Sqrt-transformed sulci depth was extracted based on the Euclidian distance between central surface and its convex hull and gyrification index was extracted based on absolute mean curvature (Luders et al., 2006). The surface-based maps were spatially registered to a surface MNI template, and spatial smoothing was applied differently for different MRI measures (as recommended in the CAT12 manual;http://www.neuro.uni-jena.de/cat12/CAT12-Manual.pdf) before statistical analysis. Complexity and gyrification maps were spatially smoothed with a 20 mm Gaussian FWHM kernel to cover both gyrus and sulcus, while sulci depth and cortical thickness maps were spatially smoothed with a 12 mm Gaussian FWHM kernel.

### 2.6 Statistical analysis of MRI data

First, we performed a restricted vertex-wise region of interest analysis within the mPFC. Second, we performed an exploratory whole cortical surface analysis. For our region of interest analysis, we created a mask by combining anatomical mPFC areas (Fo1, Fo2, Fo3, Fp1, Fp2) from The Anatomy Toolbox (Eickhoff et al., 2005,^2007^), and a 30 mm sphere centered on MNI coordinates XYZ = [−1 46 −7] (the domain-general valuation-system;Bartra et al., 2013) to “fill-in” some small gaps between the anatomical vmPFC atlas regions. This mPFC mask was transformed from volumetric to surface template MNI space with CAT12. For analyses, we used mass-univariate multiple linear regressions with log10-transformed α (risk preferences) and k (intertemporal preferences) as variables of interest, while controlling for demographic variables (i.e., age, sex, and IQ). For GMV, we also controlled total intracranial volume (TIV). Non-parametric statistical t-maps were adjusted with threshold-free cluster enhancement (TFCE) and FDR (q < .05) corrected based on 10,000 permutations using the TFCE toolbox (http://www.neuro.uni-jena.de/tfce/). We used a conjunction analysis to look for regions linked to both a greater preference for risky rewards (higher log10(α)) and a greater preference for immediate rewards (higher log10(k)). For a vertex to qualify as significant in our conjunction analysis, it had to have a significant association with both risky (log10(α)) and intertemporal (log10(k)) preferences, independently (Nichols et al., 2005).

## 3 Results

**Fig. 1.**
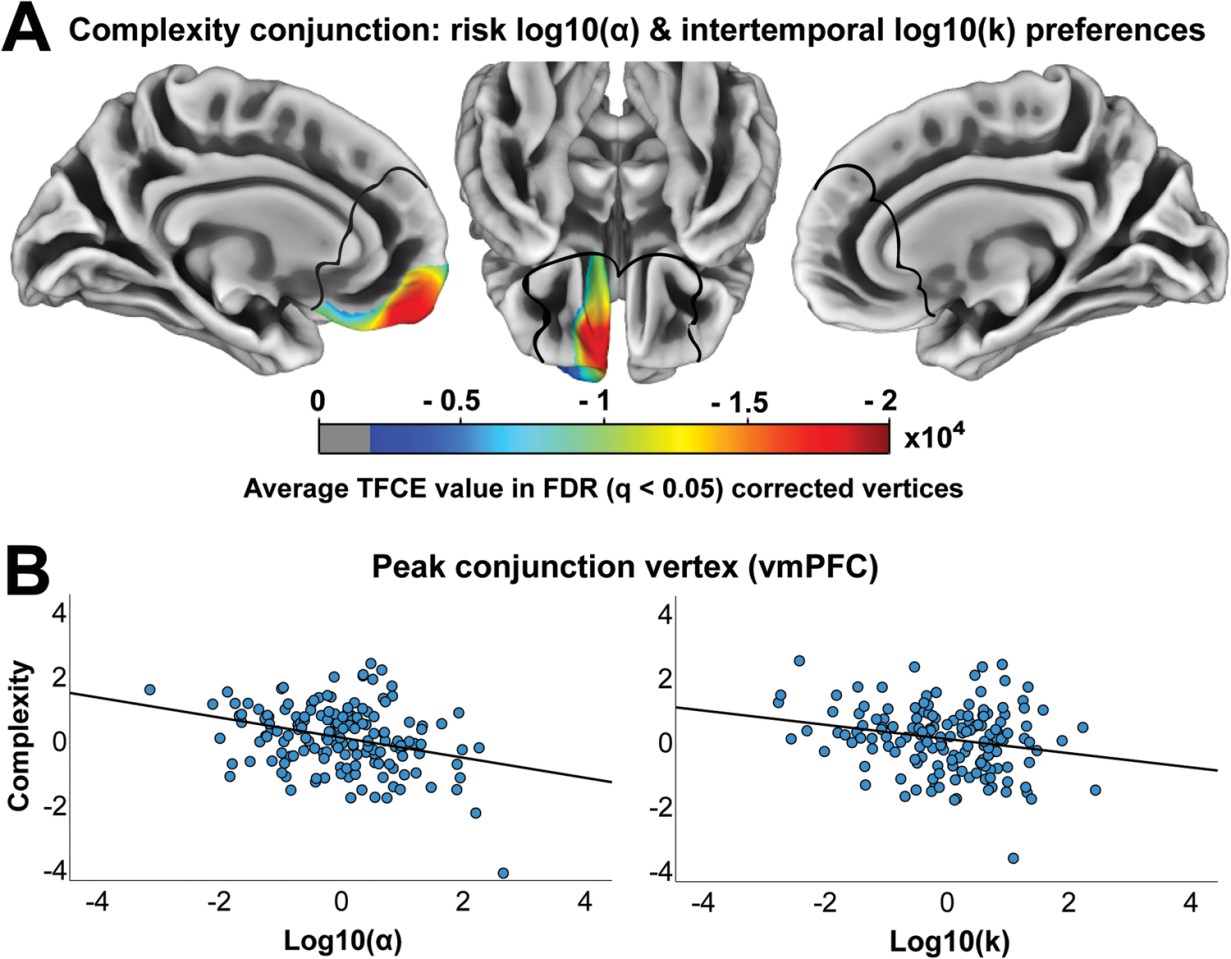
Conjunction of risk and intertemporal preferences and cortical complexity. (A) Shows the average TFCE value for vertices in which reduced cortical complexity was associated with a greater preference for risky and immediate rewards. The black lines outline the mPFC mask used to restrict the analyses to our region of interest. All results are threshold-free cluster-enhancement (TFCE) adjusted and FDR (q < .05) corrected and controlled for sex, age, IQ, and log10(α) or log10(k). (B) Scatterplots show relationships between choice preferences and cortical complexity at the peak conjunction coordinate in the ventromedial prefrontal cortex (vmPFC) (standardized residuals from regressing out age, sex, IQ, and log10(α) or log10(k)).

Behaviorally, we found the expected variability in risk and intertemporal preferences in our sample, and across the sample risk and intertemporal preferences were weakly to moderately negatively correlated, in line with the previous literature (S-Fig. 1). That is, individuals with a greater preference for risky rewards (higher risk tolerance, α) tended to have a greater preference for delayed rewards (lower delay discount rate, k). Moreover, both risk and intertemporal preferences exhibited moderately high test-retest reliability in this population. A subset of our sample (n = 121) was tested again 10 weeks later in a post-intervention session, and the correlation between the choice preferences in the baseline session we analyze below and those in the session 10 weeks later was r = 0.72 for risk and r = 0.75 for intertemporal preferences.

Though we use the model-based preference measures log10(α) and log10(k) in our analyses below, these measures were strongly correlated with model-free measures of preference. There was a strong correlation between log10(α) and the proportion of choices of the risky option (r = 0.91), as well as between log10(k) and the proportion of choices of the immediate option (r = 0.97). Using model-free measures did not qualitatively change our conjunction result. Furthermore, our measure of risk preferences, which models risk aversion using a power utility function, was strongly correlated with other measures of risk preferences calculated using different models (for an extensive comparison in these data, seeJung et al., 2018). Specifically, log10(α) was almost perfectly negatively correlated (r = -0.995) with a log10-transformed hyperbolic probability discounting parameter (M_R2_= 0.35; SE_R2_= 0.01). Thus, the associations below are likely robust to the specific measures of risk and intertemporal preferences used.

**Fig. 2.**
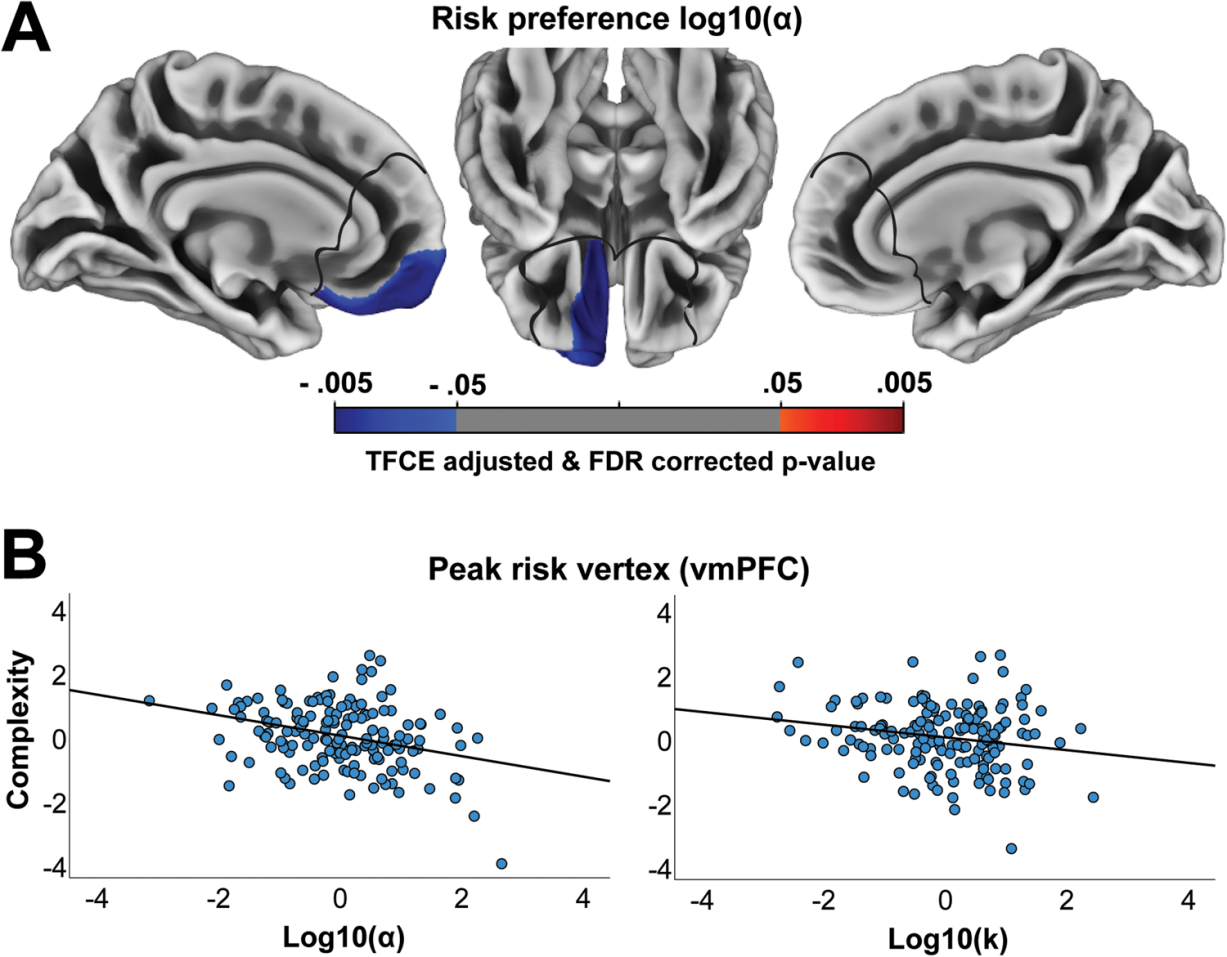
Risk preferences and cortical complexity. (A) Shows associations between cortical complexity and risk preferences. The black lines outline the mPFC mask used to restrict the analyses to our region of interest. All results are threshold-free cluster-enhancement (TFCE) adjusted and FDR (q < .05) corrected and controlled for sex, age, IQ, and log10(α) or log10(k). (B) Scatterplots show the relationships between choice preferences and cortical complexity at the peak coordinate in the ventromedial prefrontal cortex (vmPFC) for risk preferences (standardized residuals from regressing out age, sex, IQ, and log10(α) or log10(k)).

**Fig. 3.**
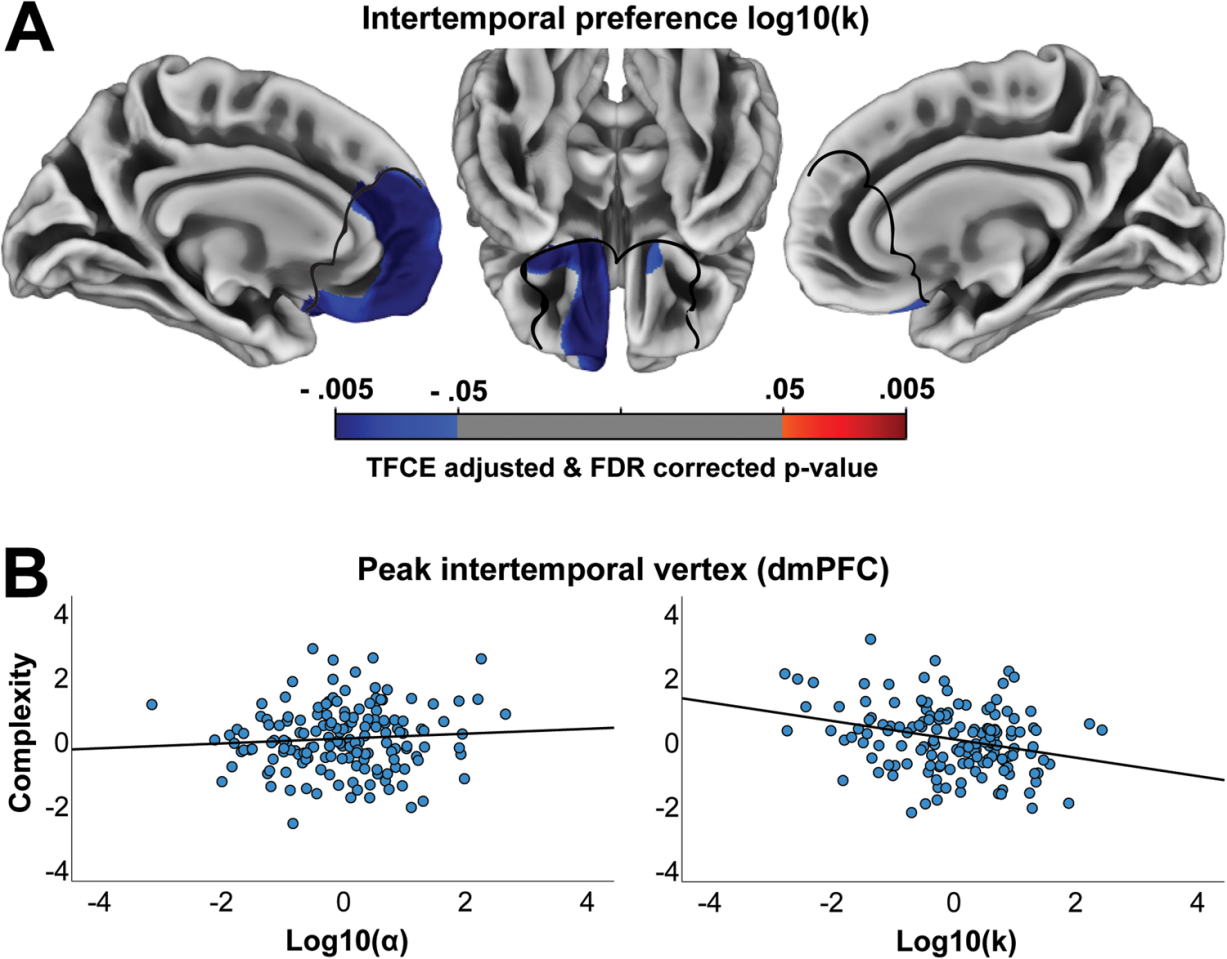
Intertemporal preferences and cortical complexity. (A) Shows associations between cortical complexity and intertemporal choice preferences. The black lines outline the mPFC mask used to restrict the analyses to our region of interest. All results are threshold-free cluster-enhancement (TFCE) adjusted and FDR (q < .05) corrected and controlled for sex, age, IQ, and log10(α) or log10(k). (B) Scatterplots show the relationships between choice preferences and cortical complexity at the peak coordinate in the dorsomedial prefrontal cortex (dmPFC) for intertemporal preferences (standardized residuals from regressing out age, sex, IQ and log10(α) or log10(k)).

Next, we investigated the relationship between cortical complexity and risk and intertemporal preferences. We found that cortical complexity in our medial frontal region of interest was associated with preferences for risky and immediate rewards. The conjunction analysis identified a region in left vmPFC, extending into the orbitofrontal cortex (OFC), where reduced cortical complexity was associated with both a greater preference for risky rewards and a greater preference for immediate rewards (Fig. 1). This association was stronger more anteriorly and bordering the frontopolar cortex. When analyzing risky preferences, there were no distinct associations in mPFC (Fig. 2A) beyond the region of left vmPFC identified in the conjunction analysis, where there was a negative association between cortical complexity and preference for risky rewards (peak MNI = [-7 46 -10], TFCE value = 21784, k = 2607). When analyzing intertemporal preferences, however, the negative association between cortical complexity and preferences for immediate rewards extended beyond the left vmPFC region identified in the conjunction (Fig. 3A) and included the dorsomedial prefrontal cortex (dmPFC; peak MNI = [-7 25 23], TFCE value = 20536, k = 6042) and right vmPFC (peak MNI = [15 15 -12], TFCE value = 3640, k = 369). To identify any regions where cortical complexity was associated with risky or intertemporal preferences beyond our region of interest, we also performed an exploratory whole-brain analysis. No results survived FDR (q < .05) correction.

**Fig. 4.**
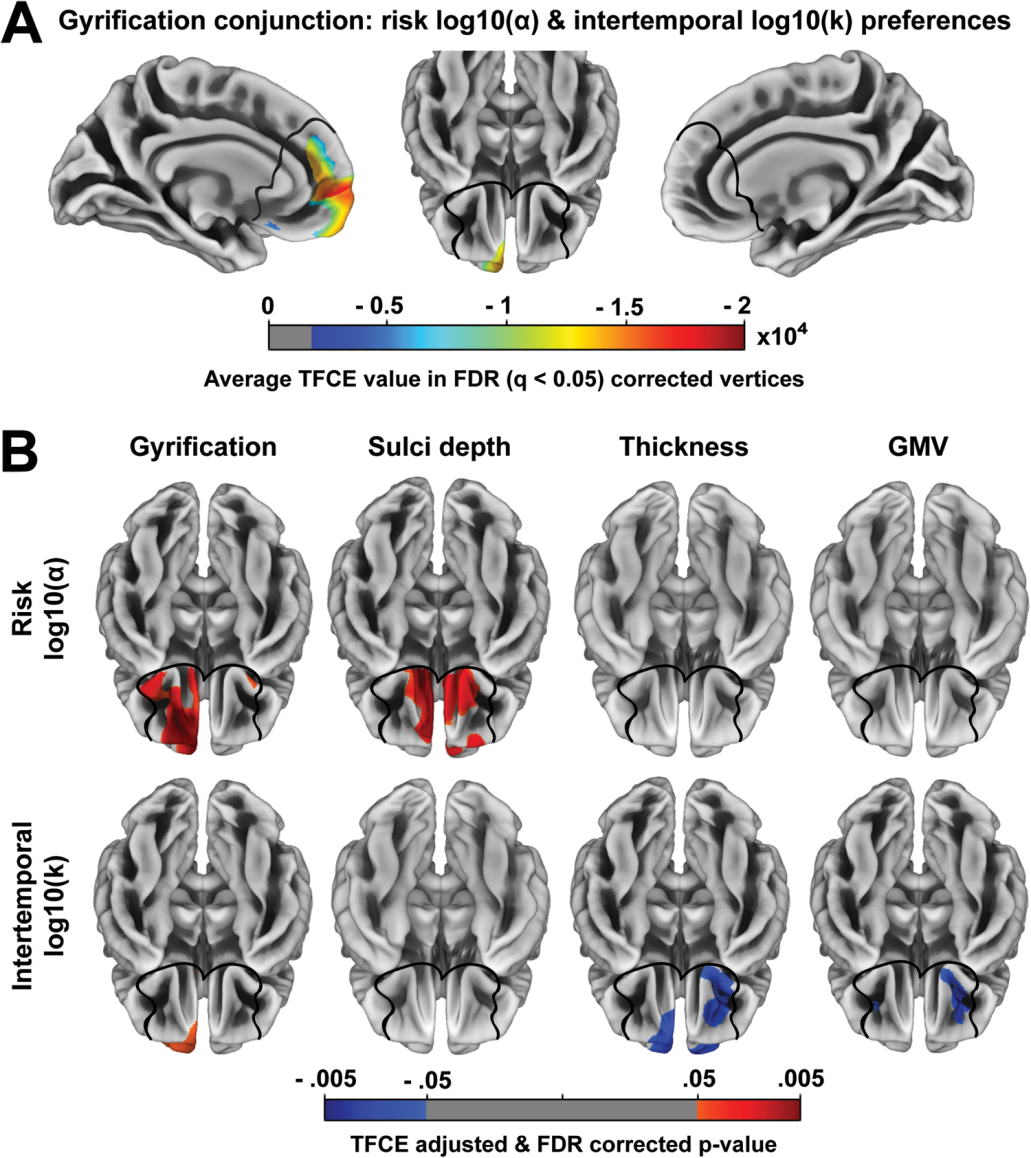
Region of interest results within mPFC for other MRI measures. (A) Shows the average TFCE value for vertices in which reduced gyrification was associated with a reduced preference for risky and immediate rewards. (B) Shows associations between gyrification, sulci depth, thickness, and grey matter volume (GMV) and risky or intertemporal preferences. The black lines outline the mPFC mask used to restrict the analyses to our region of interest. All results are threshold-free cluster-enhancement (TFCE) adjusted and FDR (q < .05) corrected and controlled for sex, age, and IQ. GMV was also controlled for total intracranial volume.

We performed several additional analyses to show that this relationship with risky and intertemporal preferences is not better explained by other structural measures, such as gyrification, sulci depth, cortical thickness, or GMV. In our dataset, cortical complexity indeed shows significant, though modestly sized, whole-brain relationships with gyrification (r(299879) = -.28, p < .001), sulci depth (r(299879) = .09, p < .001), cortical thickness (r(299879) = -.16, p < .001), and GMV (r(299879) = -.05, p < .001). However, none of these other structural measures recapitulates the relationship we found between cortical complexity and risk and intertemporal preferences in our medial frontal region of interest. The conjunction analysis only identified an overlapping association for gyrification: a region extending from left dmPFC to anterior left vmPFC, in which reduced gyrification (i.e., frequency and magnitude of cortical folding) was associated with both a reduced preference for risky rewards and a reduced preference for immediate rewards (Fig. 4A). The gyrification conjunction overlapped only slightly with the complexity conjunction in the anterior vmPFC and frontopolar cortex. The conjunction analysis did not find any significant overlapping associations for the other structural measures.

Considering risky and intertemporal preferences separately, there were several distinct associations with the other structural measures, though none of these exhibited the same pattern that we observed with cortical complexity. For gyrification, the positive association with a preference for risky rewards extended beyond the dmPFC/frontopolar conjunction area (peak MNI = [-14 26 19], TFCE value = 22146, k = 5717) to the left ventral PFC and a small cluster in the right ventral PFC (peak MNI = [27 10 11], TFCE value = 2681, k = 111), while the positive association with a preference for immediate rewards extended beyond the dmPFC/frontopolar conjunction area (peak MNI = [-13 51 12], TFCE value = 14427, k = 3527) to the left vmPFC (peak MNI = [8 25 17], TFCE value = 4643, k = 946), right frontopolar cortex (peak MNI = [-14 44 17] TFCE value = 6189, k = 562), and right dmPFC (peak MNI = [-3 20 -14], TFCE value = 5191, k = 228). For sulci depth, we found a positive association with a preference for risky rewards in the left vmPFC (peak MNI = [-3 31 -14], TFCE value = 17618, k = 2175) and right vmPFC (peak MNI = [10 19 -9], TFCE value = 5400, k = 3656), but no association between sulci depth and intertemporal preferences (Fig. 4B). For cortical thickness, we found a negative association with a preference for immediate rewards in the right vmPFC/frontopolar cortex (peak MNI = [23 31 -4], TFCE value = 6929, k = 1362), right dmPFC (peak MNI = [1 33 37], TFCE value = 5591, k = 2029), and left dmPFC (peak MNI = [-28 39 23], TFCE value = 3645, k = 239) extending to OFC and the frontopolar cortex (peak MNI = [-6 50 -5], TFCE value = 10986); but no association between cortical thickness and risk preferences (Fig. 4B). For GMV, we found a negative association with a preference for immediate rewards in the right OFC (peak MNI = [22 38 -8], TFCE value = 785, k = 944) and left OFC (peak MNI = [-22 32 -6], TFCE value = 423, k = 156), but no association between GMV and risk preferences (Fig. 4B).

Finally, the relationship between cortical complexity and risky and intertemporal preferences still holds when controlling for the other structural measures. We performed two General Linear Regression Models with risk or intertemporal preference as the dependent variable and the five structural measures, demographics, and the other choice preference (i.e., the one not used as the dependent variable) as independent variables. Each of the MRI measures were averaged across the peak conjunction area (i.e., the 500 vertices with the highest average TFCE value) roughly corresponding to the red area inFigure 1A. In these regressions, cortical complexity was still a significant predictor of risk preference (β = -.29, t(146) = -2.74, p = .007) and intertemporal preference (β = -.48, t(146) = -2.24, p = .027) when controlling for the other four MRI measures (all ps > .05). Taken together, our results show a relationship between cortical complexity in mPFC and risky and intertemporal preferences that cannot be explained as secondary to other structural measures. To potentially generate hypotheses for future studies, we present uncorrected whole-surface results for all five structural measures inS-Figure 2.

## 4 Discussion

Here, we investigated the relationship between local cortical complexity in mPFC and individual differences in preferences for risky and immediate rewards. We found that reduced cortical complexity in left vmPFC, extending into the orbitofrontal cortex and frontopolar cortex, was associated with a greater preference for both risky and immediate rewards. Furthermore, this relationship was not better explained by other structural measures that were correlated with complexity, including gyrification, sulci depth, cortical thickness, and GMV. The associations between cortical complexity and intertemporal choice extended beyond these regions, such that a preference for immediate rewards was associated with reduced cortical complexity in left dmPFC and right vmPFC. Taken together, these findings suggest that cortical complexity in vmPFC may reflect one shared aspect of individual differences in risky and intertemporal preferences.

The association between reduced cortical complexity in vmPFC and a greater preference for both risky and immediate rewards is a novel finding consistent with our expectations based on neuropsychological literature. Specifically, individuals with vmPFC lesions have a greater preference for risky and immediate rewards (Mok et al., 2021;Peters & D’Esposito, 2020). However, large lesions are not as informative as to whether shared or distinct areas in vmPFC are responsible for these effects on risky and intertemporal choices. Here, we were able to show that cortical folding properties in the same area of left vmPFC are associated with both risky and intertemporal preferences in healthy participants. Although our finding was only significant in the left hemisphere, we do not wish to draw a strong conclusion about hemispheric differences given below-threshold associations in the same direction in the right vmPFC.

Our results also contribute to a growing understanding of functional subdivisions within vmPFC. Interestingly, the region where cortical complexity is associated with risky and intertemporal preferences is anterior and ventral to the area of vmPFC where activity reflects the subjective value of choice options across domains (Bartra et al., 2013), including in risky, intertemporal, and effortful choice (Seaman et al., 2018). Several studies found that lesions to more ventral areas of vmPFC caused a stronger preference for immediate rewards (Mok et al., 2021;Peters & D’Esposito, 2016;Sellitto et al., 2010), while theFellows and Farah (2005)study that found no effect on intertemporal preferences included individuals with more dorsal vmPFC lesions. Moreover, in a large study of adolescents and young adults, a greater preference for immediate rewards was associated with reduced cortical thickness in a similar region of vmPFC to the one identified here (Pehlivanova et al., 2018). It is less clear whether there is a similar ventral-dorsal difference for risky preferences because the lesion loci and tasks vary widely across studies (Clark et al., 2008;Leland & Grafman, 2005;Levens et al., 2014;Manes et al., 2002;Spaniol et al., 2019;Studer et al., 2015;Weller et al., 2007). Nevertheless, the two studies using larger groups with focal lesions that overlap with the more anterior and ventral region of vmPFC found a greater preference for risky rewards (Clark et al., 2008;Studer et al., 2015). One possibility is therefore that the ventral and anterior aspects of vmPFC have a different function than mid-vmPFC, which seems likely given the hierarchical functional organization of the PFC and more granular structural organization from mPFC to OFC/frontopolar cortex (for review see,Wallis, 2012).

Reduced cortical complexity in vmPFC may be a structural marker for one aspect of individual differences in risky and intertemporal preferences, possibly a tendency towards “impulsive” choices, given that it is associated with a greater preference for both larger risky over smaller certain rewards and smaller immediate over larger delayed rewards. However, the idea that a tendency towards impulsive choice is the only or even the primary driver of these preferences is problematic because, as seen in our data, the most common behavioral finding is that individuals who are more risk-seeking also tend to be more patient (Johnson et al., 2020). Moreover, there are groups of people who are risk-seeking and impatient, risk-averse and impatient, or have an equal preference for risk and delay (for a critical review, seeGreen & Myerson, 2013). Risk and intertemporal preferences are therefore likely underpinned by distinct but partially overlapping neural processes. Although here we pinpoint one shared association in vmPFC with risky and intertemporal preferences, there are likely other shared and distinct brain-behavior associations (possibly in both directions) that together contribute to overall risk and intertemporal preferences. However, our findings do imply that, all else equal, if there is a lesion or other degradation of cortical complexity in vmPFC (e.g., from clinical disorders, drug use, or age), then it would lead to more risk-seeking and impatient behavior.

Consistent with the idea that risk and intertemporal preferences have both shared and distinct neural contributions, we found that the association between intertemporal preferences and cortical complexity extended beyond the left vmPFC, most notably into the left dmPFC. Although we can only speculate about the functional significance of the association in dmPFC, neural activity in the same dmPFC region has previously been associated with subjective value during intertemporal choice (Kable & Glimcher, 2007). Similarly, structural studies have found that a greater preference for immediate rewards is associated with more GMV (Cho et al., 2013) and more cortical thickness (Bernhardt et al., 2014) in dmPFC.

Risk and intertemporal preferences change over time because of age-related atrophy, environmental context (e.g., economic recession, wars, etc.), and temporary fluctuations (e.g., mood, stress, etc.), but are likely relatively stable traits based on moderately high rank-order stability across individuals over time (Chuang & Schechter, 2015;Escobar et al., 2023;Hertwig et al., 2019;Mata et al., 2018;Meier & Sprenger, 2015;Seneca et al., 2012;Zeynep Enkavi et al., 2019). We also found a moderately high correlation between the choice preferences in the baseline session we analyzed here and those 10 weeks later, in the subset of our participants (n = 121) who returned. Our use of a large number of questions (120 per task) might contribute to the somewhat higher degree of stability we observed.

It is believed that cortical complexity depends to a large extent on genetics and early neurodevelopment (Armstrong et al., 1995;Kochunov et al., 2010;Zilles et al., 1988), suggesting relative stability over the life span. However, cortical complexity is sensitive to age-related atrophy (Madan & Kensinger, 2016) and thus likely also other environmental factors known to affect brain structure. For example, grey matter volume differences in vmPFC and other brain areas have been associated with prolonged drug-use (Mackey et al., 2019) and chronic/acute stress (Arnsten, 2009). However, it is not always easy to determine the causal direction between such associative findings. Future longitudinal studies are needed to tease apart to what extent the association between cortical complexity and choice preferences is influenced by genetics, early neurodevelopment, aging, or other environmental factors. Furthermore, since our sample is limited to young adults in the US, investigating these relationships in earlier development or older aging, or cross-culturally, would be interesting future directions.

Here, we focused our investigation on cortical complexity, though complexity is thought to be a composite of several other structural properties that are also measurable, including gyrification, sulci depth, cortical thickness, and GMV. Complexity is a measure of how space-filling, self-similar, and convoluted a brain’s surface isYotter, Nenadic, et al. (2011), and higher cortical complexity has been associated with more convoluted gyral shape, higher folding frequency, deeper sulci, and thinner cortex (Im et al., 2006;Jiang et al., 2008;King et al., 2010). We therefore expected to find that complexity had a positive relationship with gyrification and sulci depth, and a negative relationship with cortical thickness. Surprisingly, surface-wide correlations show that complexity had a negative relationship with gyrification in our data, while the expected relationships were found for sulci depth (positive) and cortical thickness (negative), respectively. Moreover, region-of-interest associations revealed that local relationships in vmPFC between complexity and sulci depth and cortical thickness deviated from expectations and from surface-wide relationships. Clearly, these different structural measures have complex relationships with each other that seem to vary across the cortex. Importantly, however, our analyses show that cortical complexity in vmPFC is associated with risky and intertemporal preferences even when controlling for these other structural measures.

In conclusion, we found that reduced cortical complexity in the vmPFC was associated with a greater preference for both risky and immediate rewards. As cortical folding properties are known to depend on genetics and early neurodevelopment, this association with risky and intertemporal preferences may reflect a neural process that contributes to individual differences in impulsive risk and intertemporal preferences. Our result is consistent with previous neuropsychological findings that vmPFC lesions lead to a greater preference for risky and immediate rewards. Given that these associations occurred in the context where greater preference for risky rewards was correlated with a greater preference for delayed rewards across the population, cortical complexity in vmPFC must necessarily be one shared contributor, among multiple distinct and shared contributors, to these preferences. Indeed, we also found evidence for a distinct relationship between dmPFC cortical complexity and intertemporal preferences. Future work should further explore the relationship between cortical complexity in different brain areas (and other structural properties beyond GMV and cortical thickness) and choice behavior.

## Supplementary Material

Supplementary Material

## Data Availability

MRI and behavioral data are available at OpenNeuro (https://doi.org/10.18112/openneuro.ds002843.v1.0.1). Second-level statistical maps for all MRI measures are available at Open Science Framework (https://doi.org/10.17605/OSF.IO/926CJ). MATLAB code for estimating risk and intertemporal preferences is available at GitHub (https://github.com/sangillee/UMm).

## References

[b1] Armstrong , E. , Schleicher , A. , Omran , H. , Curtis , M. , & Zilles , K. ( 1995 ). The ontogeny of human gyrification . Cerebral Cortex , 5 ( 1 ), 56 – 63 . 10.1093/cercor/5.1.56 7719130

[b2] Arnsten , A. F. T. ( 2009 ). Stress signalling pathways that impair prefrontal cortex structure and function . Nature Reviews Neuroscience , 10 ( 6 ), 410 – 422 . 10.1038/nrn2648 19455173 PMC2907136

[b3] Ashburner , J. , & Friston , K. J. ( 2005 ). Unified segmentation . NeuroImage , 26 ( 3 ), 839 – 851 . 10.1016/j.neuroimage.2005.02.018 15955494

[b4] Bartra , O. , McGuire , J. T. , & Kable , J. W. ( 2013 ). The valuation system: A coordinate-based meta-analysis of BOLD fMRI experiments examining neural correlates of subjective value . NeuroImage , 76 , 412 – 427 . 10.1016/j.neuroimage.2013.02.063 23507394 PMC3756836

[b5] Benzion , U. , Rapoport , A. , & Yagil , J. ( 1989 ). Discount rates inferred from decisions: An experimental study . Management Science , 35 ( 3 ), 270 – 284 . 10.1287/mnsc.35.3.270

[b6] Bernhardt , B. C. , Smallwood , J. , Tusche , A. , Ruby , F. J. M. , Engen , H. G. , Steinbeis , N. , & Singer , T. ( 2014 ). Medial prefrontal and anterior cingulate cortical thickness predicts shared individual differences in self-generated thought and temporal discounting . NeuroImage , 90 , 290 – 297 . 10.1016/j.neuroimage.2013.12.040 24384154

[b7] Bjork , J. M. , Momenan , R. , & Hommer , D. W. ( 2009 ). Delay discounting correlates with proportional lateral frontal cortex volumes . Biological Psychiatry , 65 ( 8 ), 710 – 713 . 10.1016/j.biopsych.2008.11.023 19121516

[b8] Burks , S. V. , Carpenter , J. P. , Goette , L. , & Rustichini , A. ( 2009 ). Cognitive skills affect economic preferences, strategic behavior, and job attachment . Proceedings of the National Academy of Sciences of the United States of America , 106 ( 19 ), 7745 – 7750 . 10.1073/pnas.0812360106 19416865 PMC2683075

[b9] Camille , N. , Griffiths , C. A. , Vo , K. , Fellows , L. K. , & Kable , J. W. ( 2011 ). Ventromedial frontal lobe damage disrupts value maximization in humans . Journal of Neuroscience , 31 ( 20 ), 7527 – 7532 . 10.1523/JNEUROSCI.6527-10.2011 21593337 PMC3122333

[b10] Cho , S. S. , Pellecchia , G. , Aminian , K. , Ray , N. , Segura , B. , Obeso , I. , & Strafella , A. P. ( 2013 ). Morphometric correlation of impulsivity in medial prefrontal cortex . Brain Topography , 26 ( 3 ), 479 – 487 . 10.1007/s10548-012-0270-x 23274773 PMC4452220

[b11] Chuang , Y. , & Schechter , L. ( 2015 ). Stability of experimental and survey measures of risk, time, and social preferences: A review and some new results . Journal of Development Economics , 117 , 151 – 170 . 10.1016/j.jdeveco.2015.07.008 30078930 PMC6070154

[b12] Clark , L. , Bechara , A. , Damasio , H. , Aitken , M. R. F. , Sahakian , B. J. , & Robbins , T. W. ( 2008 ). Differential effects of insular and ventromedial prefrontal cortex lesions on risky decision-making . Brain , 131 ( 5 ), 1311 – 1322 . 10.1093/brain/awn066 18390562 PMC2367692

[b13] Dahnke , R. , Yotter , R. A. , & Gaser , C. ( 2013 ). Cortical thickness and central surface estimation . NeuroImage , 65 , 336 – 348 . 10.1016/j.neuroimage.2012.09.050 23041529

[b14] Drobetz , R. , Hänggi , J. , Maercker , A. , Kaufmann , K. , Jäncke , L. , & Forstmeier , S. ( 2014 ). Structural brain correlates of delay of gratification in the elderly . Behavioral Neuroscience , 128 ( 2 ), 134 – 145 . 10.1037/a0036208 24773434

[b15] Eickhoff , S. B. , Paus , T. , Caspers , S. , Grosbras , M.-H. , Evans , A. C. , Zilles , K. , & Amunts , K. ( 2007 ). Assignment of functional activations to probabilistic cytoarchitectonic areas revisited . NeuroImage , 36 ( 3 ), 511 – 521 . 10.1016/j.neuroimage.2007.03.060 17499520

[b16] Eickhoff , S. B. , Stephan , K. E. , Mohlberg , H. , Grefkes , C. , Fink , G. R. , Amunts , K. , & Zilles , K. ( 2005 ). A new SPM toolbox for combining probabilistic cytoarchitectonic maps and functional imaging data . NeuroImage , 25 ( 4 ), 1325 – 1335 . 10.1016/j.neuroimage.2004.12.034 15850749

[b17] Escobar , G. G. , Morales-Chainé , S. , Haynes , J. M. , Santoyo , C. , & Mitchell , S. H. ( 2023 ). Moderate stability among delay, probability, and effort discounting in humans . Psychological Record , 73 ( 2 ), 149 – 162 . 10.1007/s40732-023-00537-1 PMC9931166 36820275

[b18] Fellows , L. K. , & Farah , M. J. ( 2005 ). Dissociable elements of human foresight: A role for the ventromedial frontal lobes in framing the future, but not in discounting future rewards . Neuropsychologia , 43 ( 8 ), 1214 – 1221 . 10.1016/j.neuropsychologia.2004.07.018 15817179

[b19] Fellows , L. K. , & Farah , M. J. ( 2007 ). The role of ventromedial prefrontal cortex in decision making: Judgment under uncertainty or judgment per se ? Cerebral Cortex , 17 ( 11 ), 2669 – 2674 . 10.1093/cercor/bhl176 17259643

[b20] Gaser , C. , & Dahnke , R. ( 2016 ). CAT - A computational anatomy toolbox for the analysis of structural MRI data . Human Brain Mapping . Retrieved March 15, 2022, from http://www.neuro.uni-jena.de/hbm2016/GaserHBM2016.pdf 10.1093/gigascience/giae049 PMC11299546 39102518

[b21] Gilaie-Dotan , S. , Tymula , A. , Cooper , N. , Kable , J. W. , Glimcher , P. W. , & Levy , I. ( 2014 ). Neuroanatomy predicts individual risk attitudes . Journal of Neuroscience , 34 ( 37 ), 12394 – 12401 . 10.1523/JNEUROSCI.1600-14.2014 25209279 PMC4160774

[b22] Green , L. , & Myerson , J. ( 2013 ). How many impulsivities? A discounting perspective . Journal of the Experimental Analysis of Behavior , 99 ( 1 ), 3 – 13 . 10.1002/jeab.1 23344985 PMC3893105

[b23] Grubb , M. A. , Tymula , A. , Gilaie-Dotan , S. , Glimcher , P. W. , & Levy , I. ( 2016 ). Neuroanatomy accounts for age-related changes in risk preferences . Nature Communications , 7 , 13822 . 10.1038/ncomms13822 PMC5159889 27959326

[b24] Hedderich , D. M. , Bäuml , J. G. , Menegaux , A. , Avram , M. , Daamen , M. , Zimmer , C. , Bartmann , P. , Scheef , L. , Boecker , H. , Wolke , D. , Gaser , C. , & Sorg , C. ( 2020 ). An analysis of MRI derived cortical complexity in premature-born adults: Regional patterns, risk factors, and potential significance . NeuroImage , 208 , 116438 . 10.1016/j.neuroimage.2019.116438 31811902

[b25] Hertwig , R. , Wulff , D. U. , & Mata , R. ( 2019 ). Three gaps and what they may mean for risk preference . Philosophical Transactions of the Royal Society B: Biological Sciences , 374 ( 1766 ). 10.1098/rstb.2018.0140 PMC6335455 30966925

[b26] Holt , D. D. , Green , L. , & Myerson , J. ( 2003 ). Is discounting impulsive? Evidence from temporal and probability discounting in gambling and non-gambling college students . Behavioural Processes , 64 ( 3 ), 355 – 367 . 10.1016/S0376-6357(03)00141-4 14580704

[b27] Im , K. , Lee , J. M. , Yoon , U. , Shin , Y. W. , Soon , B. H. , In , Y. K. , Kwon , J. S. , & Kim , S. I. ( 2006 ). Fractal dimension in human cortical surface: Multiple regression analysis with cortical thickness, sulcal depth, and folding area . Human Brain Mapping , 27 ( 12 ), 994 – 1003 . 10.1002/hbm.20238 16671080 PMC6871396

[b28] Jiang , J. , Zhu , W. , Shi , F. , Zhang , Y. , Lin , L. , & Jiang , T. ( 2008 ). A robust and accurate algorithm for estimating the complexity of the cortical surface . Journal of Neuroscience Methods , 172 ( 1 ), 122 – 130 . 10.1016/j.jneumeth.2008.04.018 18511127

[b29] Johnson , K. L. , Bixter , M. T. , & Luhmann , C. C. ( 2020 ). Delay discounting and risky choice: Meta-analytic evidence regarding single-process theories . Judgment and Decision Making , 15 ( 3 ), 381 – 400 . 10.1017/S193029750000718X

[b30] Jung , W. H. , Lee , S. , Lerman , C. , & Kable , J. W. ( 2018 ). Amygdala functional and structural connectivity predicts individual risk tolerance . Neuron , 98 ( 2 ), 394 - 404.e4 . 10.1016/j.neuron.2018.03.019 29628186 PMC5910234

[b31] Kable , J. W. , Caulfield , M. K. , Falcone , M. , McConnell , M. , Bernardo , L. , Parthasarathi , T. , Cooper , N. , Ashare , R. , Audrain-McGovern , J. , Hornik , R. , Diefenbach , P. , Lee , F. J. , & Lerman , C. ( 2017 ). No effect of commercial cognitive training on brain activity, choice behavior, or cognitive performance . Journal of Neuroscience , 37 ( 31 ), 7390 – 7402 . 10.1523/JNEUROSCI.2832-16.2017 28694338 PMC5546110

[b32] Kable , J. W. , & Glimcher , P. W. ( 2007 ). The neural correlates of subjective value during intertemporal choice . Nature Neuroscience , 10 ( 12 ), 1625 – 1633 . 10.1038/nn2007 17982449 PMC2845395

[b33] Kable , J. W. , & Glimcher , P. W. ( 2009 ). The neurobiology of decision: Consensus and controversy . Neuron , 63 ( 6 ), 733 – 745 . 10.1016/j.neuron.2009.09.003 19778504 PMC2765926

[b34] Kable , J. W. , & Levy , I. ( 2015 ). Neural markers of individual differences in decision-making . Current Opinion in Behavioral Sciences , 5 , 100 – 107 . 10.1016/j.cobeha.2015.08.004 28413814 PMC5389668

[b35] Keren , G. , & Roelofsma , P. ( 1995 ). Immediacy and certainty in intertemporal choice . Organizational Behavior and Human Decision Processes , 63 ( 3 ), 287 – 297 . 10.1006/obhd.1995.1080

[b36] King , R. D. , Brown , B. , Hwang , M. , Jeon , T. , & George , A. T. ( 2010 ). Fractal dimension analysis of the cortical ribbon in mild Alzheimer’s disease . NeuroImage , 53 ( 2 ), 471 – 479 . 10.1016/j.neuroimage.2010.06.050 20600974 PMC2942777

[b37] Knutson , B. , & Huettel , S. A. ( 2015 ). The risk matrix . Current Opinion in Behavioral Sciences , 5 , 141 – 146 . 10.1016/j.cobeha.2015.10.012

[b38] Kochunov , P. , Glahn , D. C. , Fox , P. T. , Lancaster , J. L. , Saleem , K. , Shelledy , W. , Zilles , K. , Thompson , P. M. , Coulon , O. , Mangin , J. F. , Blangero , J. , & Rogers , J. ( 2010 ). Genetics of primary cerebral gyrification: Heritability of length, depth and area of primary sulci in an extended pedigree of Papio baboons . NeuroImage , 53 ( 3 ), 1126 – 1134 . 10.1016/j.neuroimage.2009.12.045 20035879 PMC2888833

[b39] Leland , J. W. , & Grafman , J. ( 2005 ). Experimental tests of the somatic marker hypothesis . Games and Economic Behavior , 52 ( 2 ), 386 – 409 . 10.1016/j.geb.2004.09.001

[b40] Lempert , K. M. , Mechanic-Hamilton , D. J. , Xie , L. , Wisse , L. E. M. , de Flores , R. , Wang , J. , Das , S. R. , Yushkevich , P. A. , Wolk , D. A. , & Kable , J. W. ( 2020 ). Neural and behavioral correlates of episodic memory are associated with temporal discounting in older adults . Neuropsychologia , 146 , 107549 . 10.1016/j.neuropsychologia.2020.107549 32621907 PMC7502478

[b41] Levens , S. M. , Larsen , J. T. , Bruss , J. , Tranel , D. , Bechara , A. , & Mellers , B. A. ( 2014 ). What might have been? The role of the ventromedial prefrontal cortex and lateral orbitofrontal cortex in counterfactual emotions and choice . Neuropsychologia , 54 ( 1 ), 77 – 86 . 10.1016/j.neuropsychologia.2013.10.026 24333168 PMC4319649

[b42] Levy , I. , Snell , J. , Nelson , A. J. , Rustichini , A. , & Glimcher , P. W. ( 2010 ). Neural representation of subjective value under risk and ambiguity . Journal of Neurophysiology , 103 ( 2 ), 1036 – 1047 . 10.1152/jn.00853.2009 20032238

[b43] Luders , E. , Thompson , P. M. , Narr , K. L. , Toga , A. W. , Jancke , L. , & Gaser , C. ( 2006 ). A curvature-based approach to estimate local gyrification on the cortical surface . NeuroImage , 29 ( 4 ), 1224 – 1230 . 10.1016/j.neuroimage.2005.08.049 16223589

[b44] Luders , Eileen , Narr , K. L. , Thompson , P. M. , Rex , D. E. , Jancke , L. , Steinmetz , H. , & Toga , A. W. ( 2004 ). Gender differences in cortical complexity . Nature Neuroscience , 7 ( 8 ), 799 – 800 . 10.1038/nn1277 15338563

[b45] Mackey , S. , Allgaier , N. , Chaarani , B. , Spechler , P. , Orr , C. , Bunn , J. , Allen , N. B. , Alia-Klein , N. , Batalla , A. , Blaine , S. , Brooks , S. , Caparelli , E. , Chye , Y. Y. , Cousijn , J. , Dagher , A. , Desrivieres , S. , Feldstein-Ewing , S. , Foxe , J. J. , Goldstein , R. Z. ,… Garavan , H. ( 2019 ). Mega-analysis of gray matter volume in substance dependence: General and substance-specific regional effects . American Journal of Psychiatry , 176 ( 2 ), 119 – 128 . 10.1176/appi.ajp.2018.17040415 30336705 PMC6427822

[b46] Madan , C. R. , & Kensinger , E. A. ( 2016 ). Cortical complexity as a measure of age-related brain atrophy . NeuroImage , 134 , 617 – 629 . 10.1016/j.neuroimage.2016.04.029 27103141 PMC4945358

[b47] Manes , F. , Sahakian , B. , Clark , L. , Rogers , R. , Antoun , N. , Aitken , M. , & Robbins , T. ( 2002 ). Decision-making processes following damage to the prefrontal cortex . Brain , 125 ( 3 ), 624 – 639 . 10.1093/brain/awf049 11872618

[b48] Manjón , J. V. , Coupé , P. , Martí‐Bonmatí , L. , Collins , D. L. , & Robles , M. ( 2010 ). Adaptive non-local means denoising of MR images with spatially varying noise levels . Journal of Magnetic Resonance Imaging , 31 ( 1 ), 192 – 203 . 10.1002/jmri.22003 20027588

[b49] Mata , R. , Frey , R. , Richter , D. , Schupp , J. , & Hertwig , R. ( 2018 ). Risk preference: A view from psychology . Journal of Economic Perspectives , 32 ( 2 ), 155 – 172 . 10.1257/jep.32.2.155 30203934

[b50] Meier , S. , & Sprenger , C. D. ( 2015 ). Temporal stability of time preferences . Review of Economics and Statistics , 97 ( 2 ), 273 – 286 . 10.1162/REST_a_00433

[b51] Mohr , P. N. C. , Biele , G. , & Heekeren , H. R. ( 2010 ). Neural processing of risk . Journal of Neuroscience , 30 ( 19 ), 6613 – 6619 . 10.1523/JNEUROSCI.0003-10.2010 20463224 PMC6632558

[b52] Mok , J. N. Y. , Green , L. , Myerson , J. , Kwan , D. , Kurczek , J. , Ciaramelli , E. , Craver , C. F. , & Rosenbaum , R. S. ( 2021 ). Does ventromedial prefrontal cortex damage really increase impulsiveness? Delay and probability discounting in patients with focal lesions . Journal of Cognitive Neuroscience , 33 ( 9 ), 1909 – 1927 . 10.1162/jocn_a_01721 PMC8924794 34232999

[b53] Myerson , J. , Green , L. , Scott Hanson , J. , Holt , D. D. , & Estle , S. J. ( 2003 ). Discounting delayed and probabilistic rewards: Processes and traits . Journal of Economic Psychology , 24 ( 5 ), 619 – 635 . 10.1016/S0167-4870(03)00005-9

[b54] Nenadic , I. , Yotter , R. A. , Dietzek , M. , Langbein , K. , Sauer , H. , & Gaser , C. ( 2017 ). Cortical complexity in bipolar disorder applying a spherical harmonics approach . Psychiatry Research - Neuroimaging , 263 , 44 – 47 . 10.1016/j.pscychresns.2017.02.007 28324693

[b55] Nenadic , I. , Yotter , R. A. , Sauer , H. , & Gaser , C. ( 2014 ). Cortical surface complexity in frontal and temporal areas varies across subgroups of schizophrenia . Human Brain Mapping , 35 ( 4 ), 1691 – 1699 . 10.1002/hbm.22283 23813686 PMC6869458

[b56] Nichols , T. , Brett , M. , Andersson , J. , Wager , T. , & Poline , J. B. ( 2005 ). Valid conjunction inference with the minimum statistic . NeuroImage , 25 ( 3 ), 653 – 660 . 10.1016/j.neuroimage.2004.12.005 15808966

[b57] Owens , M. M. , Gray , J. C. , Amlung , M. T. , Oshri , A. , Sweet , L. H. , & MacKillop , J. ( 2017 ). Neuroanatomical foundations of delayed reward discounting decision making . NeuroImage , 161 , 261 – 270 . 10.1016/j.neuroimage.2017.08.045 28843539 PMC5895082

[b58] Pehlivanova , M. , Wolf , D. H. , Sotiras , A. , Kaczkurkin , A. N. , Moore , T. M. , Ciric , R. , Cook , P. A. , de La Garza , A. G. , Rosen , A. F. G. , Rupare , K. , Sharma , A. , Shinohara , R. T. , Roalf , D. R. , Gur , R. C. , Davatzikos , C. , Gur , R. E. , Kable , J. W. , & Satterthwaite , T. D. ( 2018 ). Diminished cortical thickness is associated with impulsive choice in adolescence . Journal of Neuroscience , 38 ( 10 ), 2471 – 2481 . 10.1523/JNEUROSCI.2200-17.2018 29440536 PMC5858592

[b59] Peters , J. , & Büchel , C. ( 2011 ). The neural mechanisms of inter-temporal decision-making: Understanding variability . Trends in Cognitive Sciences , 15 ( 5 ), 227 – 239 . 10.1016/j.tics.2011.03.002 21497544

[b60] Peters , J. , & D’Esposito , M. ( 2016 ). Effects of medial orbitofrontal cortex lesions on self-control in intertemporal choice . Current Biology , 26 ( 19 ), 2625 – 2628 . 10.1016/j.cub.2016.07.035 27593380

[b61] Peters , J. , & D’Esposito , M. ( 2020 ). The drift diffusion model as the choice rule in inter-temporal and risky choice: A case study in medial orbitofrontal cortex lesion patients and controls . PLoS Computational Biology , 16 ( 4 ), 1 – 25 . 10.1371/journal.pcbi.1007615 PMC7192518 32310962

[b62] Quan , P. , He , L. , Mao , T. , Fang , Z. , Deng , Y. , Pan , Y. , Zhang , X. , Zhao , K. , Lei , H. , Detre , J. A. , Kable , J. W. , & Rao , H. ( 2022 ). Cerebellum anatomy predicts individual risk-taking behavior and risk tolerance . NeuroImage , 254 , 119148 . 10.1016/j.neuroimage.2022.119148 35346839 PMC9680915

[b63] Rajapakse , J. C. , Giedd , J. N. , & Rapoport , J. L. ( 1997 ). Statistical approach to segmentation of single-channel cerebral MR images . IEEE Transactions on Medical Imaging , 16 ( 2 ), 176 – 186 . 10.1109/42.563663 9101327

[b64] Schwartz , D. L. , Mitchell , A. D. , Lahna , D. L. , Luber , H. S. , Huckans , M. S. , Mitchell , S. H. , & Hoffman , W. F. ( 2010 ). Global and local morphometric differences in recently abstinent methamphetamine-dependent individuals . NeuroImage , 50 ( 4 ), 1392 – 1401 . 10.1016/j.neuroimage.2010.01.056 20096794 PMC3478236

[b65] Seaman , K. L. , Brooks , N. , Karrer , T. M. , Castrellon , J. J. , Perkins , S. F. , Dang , L. C. , Hsu , M. , Zald , D. H. , & Samanez-Larkin , G. R. ( 2018 ). Subjective value representations during effort, probability and time discounting across adulthood . Social Cognitive and Affective Neuroscience , 13 ( 5 ), 449 – 459 . 10.1093/scan/nsy021 29618082 PMC6007391

[b66] Sellitto , M. , Ciaramelli , E. , & Di Pellegrino , G. ( 2010 ). Myopic discounting of future rewards after medial orbitofrontal damage in humans . Journal of Neuroscience , 30 ( 49 ), 16429 – 16436 . 10.1523/JNEUROSCI.2516-10.2010 21147982 PMC6634874

[b67] Seneca , N. , Wang , T. , Thompson , E. , & Kable , J. W. ( 2012 ). Normative arguments from experts and peers reduce delay discounting . Judgment and Decision Making , 7 ( 5 ), 566 – 589 . 10.1017/s1930297500006306 PMC3626281 23596504

[b68] Spaniol , J. , Di Muro , F. , & Ciaramelli , E. ( 2019 ). Differential impact of ventromedial prefrontal cortex damage on “hot” and “cold” decisions under risk . Cognitive, Affective & Behavioral Neuroscience , 19 ( 3 ), 477 – 489 . 10.3758/s13415-018-00680-1 30535630

[b69] Studer , B. , Manes , F. , Humphreys , G. , Robbins , T. W. , & Clark , L. ( 2015 ). Risk-sensitive decision-making in patients with posterior parietal and ventromedial prefrontal cortex injury . Cerebral Cortex , 25 ( 1 ), 1 – 9 . 10.1093/cercor/bht197 23926113 PMC4259274

[b70] Tohka , J. , Zijdenbos , A. , & Evans , A. ( 2004 ). Fast and robust parameter estimation for statistical partial volume models in brain MRI . NeuroImage , 23 ( 1 ), 84 – 97 . 10.1016/j.neuroimage.2004.05.007 15325355

[b71] Wallis , J. D. ( 2012 ). Cross-species studies of orbitofrontal cortex and value-based decision-making . Nature Neuroscience , 15 ( 1 ), 13 – 19 . 10.1038/nn.2956 PMC3549638 22101646

[b72] Weller , J. A. , Levin , I. P. , Shiv , B. , & Bechara , A. ( 2007 ). Neural correlates of adaptive decision making for risky gains and losses . Psychological Science , 18 ( 11 ), 958 – 964 . 10.1111/j.1467-9280.2007.02009.x 17958709

[b73] Yotter , R. A. , Dahnke , R. , Thompson , P. M. , & Gaser , C. ( 2011 ). Topological correction of brain surface meshes using spherical harmonics . Human Brain Mapping , 32 ( 7 ), 1109 – 1124 . 10.1002/hbm.21095 20665722 PMC6869946

[b74] Yotter , R. A. , Nenadic , I. , Ziegler , G. , Thompson , P. M. , & Gaser , C. ( 2011 ). Local cortical surface complexity maps from spherical harmonic reconstructions . NeuroImage , 56 ( 3 ), 961 – 973 . 10.1016/j.neuroimage.2011.02.007 21315159

[b75] Yu , L. Q. , Dana , J. , & Kable , J. W. ( 2022 ). Individuals with ventromedial frontal damage display unstable but transitive preferences during decision making . Nature Communications , 13 ( 1 ), 1 – 10 . 10.1038/s41467-022-32511-w PMC9376076 35963856

[b76] Yu , L. Q. , Kan , I. P. , & Kable , J. W. ( 2020 ). Beyond a rod through the skull: A systematic review of lesion studies of the human ventromedial frontal lobe . Cognitive Neuropsychology , 37 ( 1–2 ), 97 – 141 . 10.1080/02643294.2019.1690981 31739752

[b77] Zeynep Enkavi , A. , Eisenberg , I. W. , Bissett , P. G. , Mazza , G. L. , MacKinnon , D. P. , Marsch , L. A. , & Poldrack , R. A. ( 2019 ). Large-scale analysis of test–retest reliabilities of self-regulation measures . Proceedings of the National Academy of Sciences of the United States of America , 116 ( 12 ), 5472 – 5477 . 10.1073/pnas.1818430116 30842284 PMC6431228

[b78] Zilles , K. , Armstrong , E. , Schleicher , A. , & Kretschmann , H. J. ( 1988 ). The human pattern of gyrification in the cerebral cortex . Anatomy and Embryology , 179 ( 2 ), 173 – 179 . 10.1007/BF00304699 3232854

