# Supplementary material for: Reduced cortical complexity in ventromedial prefrontal cortex is associated with a greater preference for risky and immediate rewards
Source: Imaging Neurosci (Camb). 2024 Apr 18;2:imag-2-00143. doi: 10.1162/imag_a_00143 (PMC12247602; doi:10.1162/imag_a_00143)
Supplement: Supplementary Material [file imag_a_00143-supp.pdf]

Supplemental Material: Reduced cortical complexity in ventromedial prefrontal cortex is associated with a greater preference for risky and immediate rewards

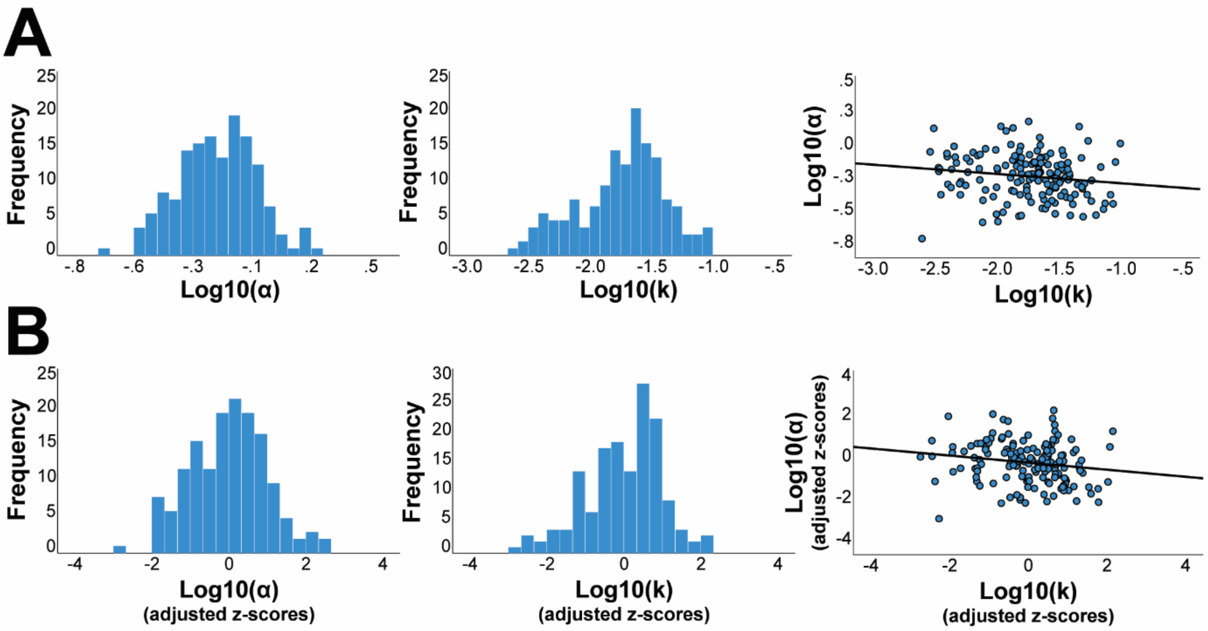

S-Figure 1. Behavioral measures. (**A**) Histograms and scatter plot of log10(α) and log10(k). (**B**) Histograms and a scatter plot with standardized residuals of log10(α) and log10(k) after regressing out age, sex, IQ.

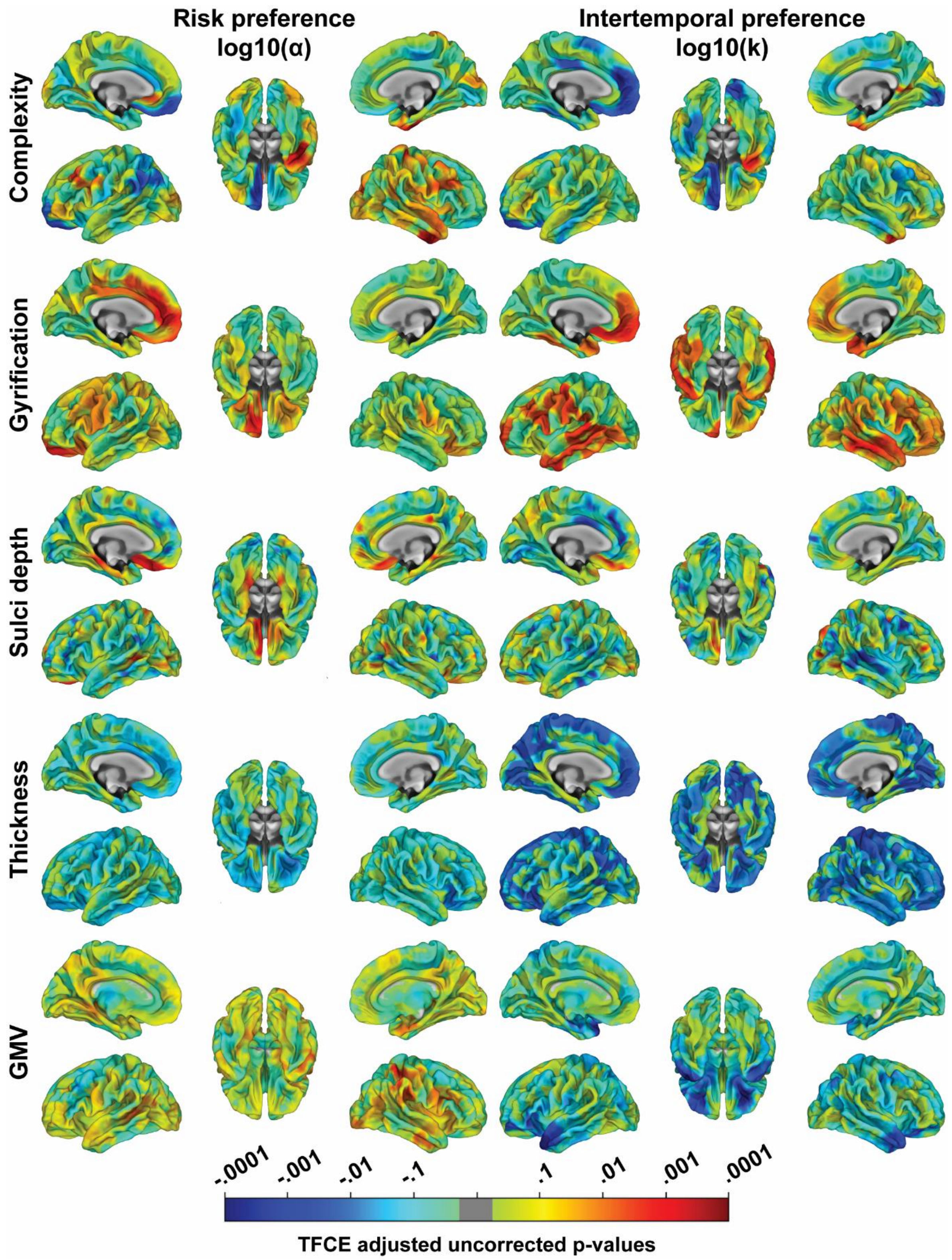

S-Figure 2. TFCE adjusted uncorrected whole-surface results. Shows associations between all five MRI measures and risk or intertemporal preference. All results are threshold-free cluster-enhancement (TFCE) adjusted, uncorrected for multiple comparisons, and

controlled for sex, age, and IQ. Grey matter volume (GMV) was also controlled for total intracranial volume.
